# Supplementary material for: Concordance of p16INK4a and E6*I mRNA among HPV-DNA-Positive Oropharyngeal, Laryngeal, and Oral Cavity Carcinomas from the ICO International Study
Source: Cancers (Basel). 2022 Aug 4;14(15):3787. doi: 10.3390/cancers14153787 (PMC9367257; doi:10.3390/cancers14153787)
Supplement: Supplementary file 1 [file cancers-14-03787-s001.zip › cancers-1822156-supplementary.pdf]

**Figure S1. Number of HPV-DNA positive head and neck cancer samples included in the study, by region**

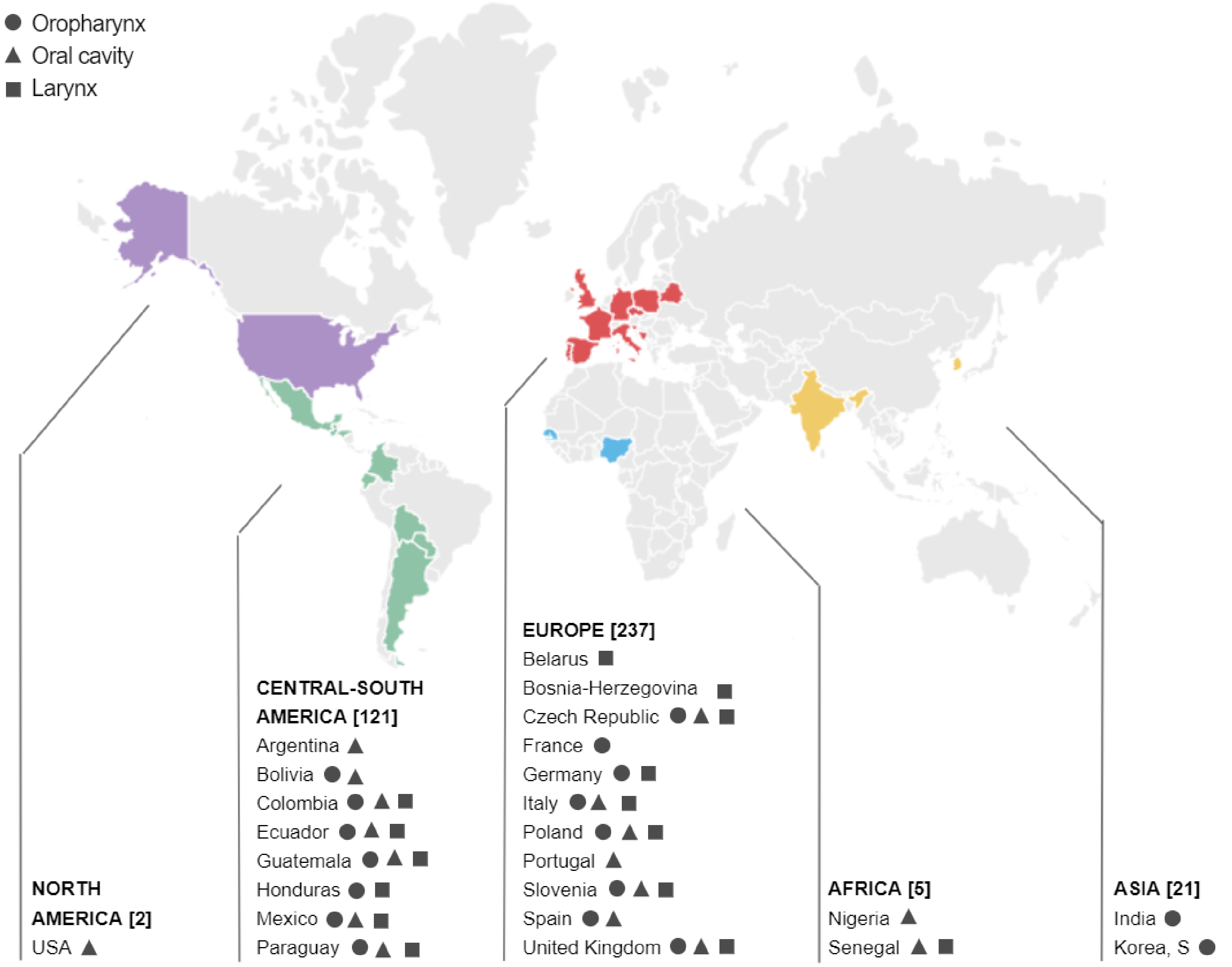

**Table S1. Descriptive characteristics of HPV-DNA positive head and neck cancer patients included in the analysis**

| Characteristics | Total (n=386) No. (%) | Oropharynx (n=257) No. (%) | Oral cavity (n=78) No. (%) | Larynx (n=51) No. (%) |
|---|---|---|---|---|
| Geographical origin | | | | |
| Europe | 237 (61.4) | 174 (67.7) | 40 (51.3) | 23 (45.1) |
| North America | 2 (0.5) | 0 (0.0) | 2 (2.6) | 0 (0.0) |
| Central-South America | 121 (31.3) | 62 (24.1) | 34 (43.6) | 25 (49.0) |
| Africa | 5 (1.3) | 0 (0.0) | 2 (2.6) | 3 (5.9) |
| Asia | 21 (5.4) | 21 (8.2) | 0 (0.0) | 0 (0.0) |
| Sex | | | | |
| Male | 276 (71.5) | 188 (73.2) | 49 (62.8) | 39 (76.5) |
| Female | 109 (28.1) | 68 (26.5) | 29 (37.2) | 12 (23.5) |
| Missing | 1 (0.3) | 1 (0.4) | 0 (0.0) | 0 (0.0) |
| Year of diagnosis | | | | |
| 1990-1994 | 7 (1.8) | 7 (2.7) | 0 (0.0) | 0 (0.0) |
| 1995-1999 | 18 (4.7) | 13 (5.1) | 5 (6.4) | 0 (0.0) |
| 2000-2004 | 58 (15.0) | 43 (16.7) | 9 (11.5) | 6 (11.8) |
| 2005-2009 | 214 (55.4) | 128 (49.8) | 54 (69.2) | 32 (62.7) |
| 2010-2012 | 89 (23.1) | 66 (25.7) | 10 (12.8) | 13 (25.5) |
| Range | 1991-2012 | 1991-2012 | 1995-2012 | 2000-2012 |
| Age at diagnosis, y | | | | |
| ≤53 | 133 (34.5) | 90 (35.0) | 22 (28.2) | 21 (41.2) |
| 54-61 | 100 (25.9) | 77 (30.0) | 11 (14.1) | 12 (23.5) |
| 62-70 | 83 (21.5) | 52 (20.2) | 21 (26.9) | 10 (19.6) |
| ≥71 | 66 (17.1) | 38 (14.8) | 20 (25.6) | 8 (15.7) |
| Missing | 4 (1.0) | 0 (0.0) | 4 (5.1) | 0 (0.0) |
| Mean (SD) | 58.5 (12.7) | 58.5 (11.7) | 60.2 (14.9) | 55.8 (14.0) |
| Age range | 19-91 | 26-91 | 19-85 | 19-85 |
| Histological diagnosis | | | | |
| SCC NOS/conventional non keratinizing | 146 (37.8) | 109 (42.4) | 23 (29.5) | 14 (27.5) |
| Conventional keratinizing | 137 (35.5) | 60 (23.3) | 43 (55.1) | 34 (66.7) |
| Conventional exophytic keratinizing | 4 (1.0) | 3 (1.2) | 1 (1.3) | 0 (0.0) |
| Basaloid/papillary | 97 (25.1) | 83 (32.3) | 11 (14.1) | 3 (5.9) |
| Undifferentiated carcinoma | 1 (0.3) | 1 (0.4) | 0 (0.0) | 0 (0.0) |
| Adenosquamous carcinoma | 1 (0.3) | 1 (0.4) | 0 (0.0) | 0 (0.0) |

**Members of the ICO International HPV in Head and Neck Cancer Study Group**

Argentina: Estela Irene Albanesi (Ministerio de Salud de la Provincia de Buenos Aires); Virginia Estrada, Sandra Erzi (Hospital San Juan de Dios); Susana Miciquevich (Facultad de Odontología de la UNLP);

Bangladesh: Ashrafun Nessa, AJE Nahar Rahman, Mohammed Kamal (Bangabandhu Sheikh Mujib Medical University – BSMMU); Faruk Ahmed (Dhaka Medical College Hospital);

Belarus: Halina Viarheichyk, Sitnikov Valeriy (Gomel State Medical University); Achynovich Searhei (Gomel Regional Clinical Oncological Hospital);

Bolívia: Edith Claros, Pedro A. Vega, Jannette Mollinedo (Servicio de Patología Instituto Oncológico del Oriente Biliviano (IOOB); Laboratorio Privado de Patología Oncos); Pablo Sitic Vargas, Lily Marcela Márquez (Servicio de Patología Instituto Oncológico del Oriente Biliviano (IOOB));

Bosnia & Herzegovina: Ermina Iljazovic (Pathology Department; University Clinical Center Tuzla; BiH);

Chile: Rodrigo Prado, Carla Molina, Rosa Muñoz (Centro de Oncología Preventiva, Facultad de Medicina, Universidad de Chile); Ximena Rodriguez, Marisol Guerrero, Virginia Leiva, Elsa Olave, Claudia Ramis, Viviana Toro (Hospital de San José);

Colombia: Enrique Cadena, Raúl Murillo, Gustavo Adolfo Hernández Suárez, Carlos Eduardo Pinzón (Instituto Nacional de Cancerología); Manuel Enrique González, Sandra Aruachan, Marcos Torregrossa (Instituto Médico de Alta Tecnología, IMAT Oncomédica, Monteria);

Czech Republic: Václav Mandys (3rd Faculty of Medicine, Charles University and University Hospital King's Wineyards); Jan Laco (Charles University Faculty of Medicine and University Hospital Hradec Kralove);

Ecuador: Leopoldo Tinoco (Hospital Oncológico Solca-Quito); Juan Carlos Ruíz Cabezas, Fernando Camacho, Rina Quinto, Leyda Jaramillo (Laboratorio Clínico y Molecular, Hospital de SOLCA Matriz Guayaquil);

France: Valerie Costes, Renaoud Garre, Anne Sophie Ramayl (Hospital Gui de Chauliac-Department de Pathologie); Massimo Tommasino, Tarik Gheit (International Agency for Research on Cancer);

Germany: Michael Pawlita, Gordana Halec, Dana Holzinger (Department Genome Changes and Carcinogenesis, Heildelberg); Falko Fend, Thorsten Biegner (Institute of Pathology and Neuropathology and Comprehensive Cancer Center,University Hospital Tuebingen);

Guatemala: Edgar Kestler , Luis Estuardo Lombardi, Obdulia Salic, Sergio Marroquin, Victor Argueta, Sergio Marroquin (Centro de Investigación Epidemiológica en Salud Sexual y Reproductiva - CIESAR, Hospital General San Juan de Dios), Walter Guerra (Instituto de Cancerología, INCA); Hesler Morales Mérida, Marcos Mauricio Siliezar Tala (Instituto de Cancerologia “Dr. Bernardo del Valle S” );

Honduras: Annabelle Ferrera (Escuela de Microbiología, Universidad Nacional Autónoma de Honduras); Flora Duarte Muñoz, Marco Molinero, Luxely Toledo, Eda Sofía Calix, Nelly Sarai Padilla (Centro de Cáncer Emma Romero de Callejas, Tegucigalpa, Honduras);

India: Asha Jain (Cancer Prevention and Relief Society Raipur); Sushil K Giri (Regional Cancer Center, Cuttack); Maheep Bhalla (JLN Hospital & Research Center,BSP, Bhilai); Bharat Patel (Lab One Raipur); PSA Sarma (BSP Hospital); Ravi Mehrotra (M.L.N Medical College, Allahabad; Institute of Cytology and Preventive Oncology (ICMR), Uttar Pradesh); Mamta Singh (M.L.N Medical College, Allahabad);

Italy: Maria Benevolo, Renato Covello, Giuseppe Spriano, Barbara Pichi (Regina Elena National Cancer Istitute), Maria Gabriella Donà (San Gallicano Dermatologic Institute);

Korea: Hai-Rim Shin (Western Pacific Regional Office, World Health Organization); Jin-Kyoung Oh (National Cancer Center); Jung-il Suh (National Medical Center); Seo-Hee Rha (Dong-A University Hospital); Dong-chul Kim (Kangnam St. Mary's Hospital); Kang Chang-Suk (Yeoui St. Mary's Hospital);

Mexico: Isabel Alvarado-Cabrero (Instituto Mexicano del Seguro Social); Patricia Alonso Viveros (Pathology Department of Hospital General de México; Instituto Nacional de salud Pública), Marco Antonio Duran Padilla, Maria Esther Gutiérrez Díaz Ceballos (Pathology Department of Hospital General de México; Faculty of Medicine Universidad Nacional Autónoma de México); Claudia Magaña-León, Rubén López-Revilla (Instituto Potosino de Investigación Científica y Tecnológica, AC); Cuauhtémoc Oros (Hospital Central Ignacio Morones Prieto, San Luis Potosí); Marco Antonio Duran Padilla, Patricia Alonso Viveros, María Esther Gutiérez Díaz Ceballos (HGM Unidad 310 (Patología), Col Doctores, Mexico DF, Delegación Cauhutémoc);

Nigeria: A.A.F. Banjo, F.B. Abdulkareem, A.O. Daramola, C.C. Anunobi, R.U. Anorlu (Lagos University Teaching Hospital Idi-Araba); Oluseyi Folake Ajayi (Department of Oral Pathology, College of Medicine, University of Lagos, Lagos, Nigeria); Sani Malami, Ali Bala Umar (Faculty of Medicine, Bayero University);

Paraguay: Elena Kasamatsu, Antonio Leoploldo Cubilla, Francisco Perrota, Susy Figueredo, (Instituto de Investigaciones en Ciencias de la Salud, Universidad Nacional de Asunción);

Philippines: Efren Javier Domingo, Jericho Thaddeus P. Luna, Maria Julieta V. Germar, Arnold M. Fernandez, Carolyn Zalameda Castro, Roslyn Balacuit (University of the Philippine College of Medicine General Hospital);

Poland: Tomasz Szafaroswski (Ent Department, Devision of Medicine and Dentistry, Warsaw Medical University, Czerniakowski Hospital);

Portugal: Ana Felix, Jorge Manuel Soares (Instituto Portugues de Oncologia de Lisboa Francisco Gentil);

Senegal: Cathy Ndiaye, Nafissatou Ndiaye Ba, Victorino Mendes (HOGGY stands for Hôpital Général de Grand Yoff ; DANTEC - Hôpital A. Le Dantec; FAC - Faculté de Médecine - Université Cheikh A. Diop);

Slovenia: Mario Poljak, Boštjan J. Kocjan, Nina Gale, Polona J. Maver (University of Ljubljana, Faculty of Medicine, Institute of Microbiology and Immunology, Ljubljana, Slovenia);

Spain: Belén Lloveras, Gemma Martín (Hospital del Mar); Llúcia Alós (Hospital Clínic, Universidad de Barcelona); Julio Velasco (Hospital San Agustín); Maria Alejo, Anna Mulero (Hospital General de l'Hospitalet); Xavier León, Miquel Quer (Hospital de Sant Pau); Marcial García-Rojo, Lucía González López, Matías Cuesta Gil (Hospital General Universitario de Ciudad Real); Laia Alemany, Francesc Xavier Bosch, Ignacio G. Bravo, Vanesa Camón, Xavier Castellsagué, Omar Clavero, Silvia de Sanjosé, Anna Esteban, Yolanda Florencia, Joellen Klaustermeier, Marisa Mena, Nubia Muñoz, Beatriz Quirós, Cristina Rajo, Sara Tous, Marleny Vergara (IDIBELL, Institut Català d'Oncologia-Catalan Institute of Oncology);

The Netherlands: Wim G.V. Quint, Anco C. Molijn, Daan T. Geraets, Núria Guimerà (DDL Diagnostic Laboratory); Chris J.L.M Meijer (Vrije Universiteit Medical Center);

Turkey: Alp Usubutun, Arzu Ruacan (Hacettepe University); Arzu Ruacan (Koc University School of Medicine)

UK: Henry Kitchener, Gillian Hall (School of Medicine, University of Manchester); Godfrey Wilson (Manchester Royal Infirmary); Mehanna Hisham, Davy Rapozo (University of Brimingham), Whitmore Shelley, James Sean (UHCW NHS Trust);

USA: Wendy Cozen, Brenda Y. Hernández, Charles Lynch, Daniel B. Olson, Freda R. Selk (Cancer Center, Hawaii-Iowa); Marc T. Goodman (Cancer Center, Hawaii-Iowa; Cedars Sinai Medical Center, Los Angeles, California, USA); Edyta C. Pirog (New York Hospital - Cornell Medical Centre);

Venezuela: Pablo Dabed (Universidad Central de Venezuela).

The advisory committee members are: Chris J Meijer, Massimo Tommasino, Michael Pawlita, Wim Quint, Llúcia Alós, and Nubia Muñoz.
